# On a new discrete Mulholland-type inequality in the whole plane

**DOI:** 10.1186/s13660-018-1777-9

**Published:** 2018-07-24

**Authors:** Bicheng Yang, Qiang Chen

**Affiliations:** 1 Department of Mathematics, Guangdong University of Education, Guangzhou, P.R. China; 2 Department of Computer Science, Guangdong University of Education, Guangzhou, P.R. China

**Keywords:** 26D15, 47A07, Mulholland-type inequality, Parameter, Weight coefficient, Equivalent form, Operator expression

## Abstract

A new discrete Mulholland-type inequality in the whole plane with a best possible constant factor is presented by introducing multi-parameters, applying weight coefficients, and using Hermite–Hadamard’s inequality. Moreover, the equivalent forms, some particular cases, and the operator expressions are considered.

## Introduction

Assume that $p > 1$, $\frac{1}{p} + \frac{1}{q} = 1$, $a_{m},b_{n} \ge0$, $0 < \sum_{m = 1}^{\infty} a_{m}^{p} < \infty$, and $0 < \sum_{n = 1}^{\infty} b_{n}^{q} < \infty$, Hardy–Hilbert’s inequality is provided as follows (cf. [1]):

1 $$ \sum_{n = 1}^{\infty} \sum _{m = 1}^{\infty} \frac{a_{m}b_{n}}{m + n} < \frac{\pi}{\sin(\pi/p)} \Biggl( \sum_{m = 1}^{\infty} a_{m}^{p} \Biggr)^{\frac{1}{p}} \Biggl( \sum_{n = 1}^{\infty} b_{n}^{q} \Biggr)^{\frac{1}{q}}, $$

where $\frac{\pi}{\sin(\pi/p)}$ is the best possible constant factor. By Theorem 343 in [1] (replacing $\frac{a_{m}}{m} $ and $\frac {b_{n}}{n} $ by $a _{m}$ and $b _{n}$, respectively), it yields the following Mulholland’s inequality:

2 $$ \sum_{n = 2}^{\infty} \sum _{m = 2}^{\infty} \frac{a_{m}b_{n}}{\ln mn} < \frac{\pi}{\sin(\pi/p)} \Biggl( \sum_{m = 2}^{\infty} \frac{a_{m}^{p}}{m} \Biggr)^{\frac{1}{p}} \Biggl( \sum_{n = 2}^{\infty} \frac{b_{n}^{q}}{n} \Biggr)^{\frac{1}{q}}. $$

Equations (1) and (2) are important inequalities in analysis and its applications (cf. [1, 2]).

In 2007, Yang [3] firstly provided the following Hilbert-type integral inequality in the whole plane:

3 $$ \int_{ - \infty}^{\infty} \int_{ - \infty}^{\infty} \frac{f(x)g(y)}{(1 + e^{x + y})^{\lambda}} \,dx\,dy < B\biggl( \frac{\lambda}{2},\frac{\lambda}{ 2}\biggr) \biggl( \int_{ - \infty}^{\infty} e^{ - \lambda x}f^{2}(x)\,dx \int_{ - \infty}^{\infty} e^{ - \lambda y}g^{2}(y)\,dy \biggr)^{\frac{1}{2}}, $$

where $B(\frac{\lambda}{2},\frac{\lambda}{2})$ ($\lambda> 0$) is the best possible constant factor. Various extensions of (1)–(3) have been presented since then (cf. [4–15]).

Recently, Yang and Chen [16] presented an extension of (1) in the whole plane as follows:

4 $$ \begin{aligned}[b] &\sum_{ \vert n \vert = 1}^{\infty} \sum_{ \vert m \vert = 1}^{\infty} \frac{a_{m}b_{n}}{( \vert m - \xi \vert + \vert n - \eta \vert )^{\lambda}} \\&\quad< 2B(\lambda_{1},\lambda_{2}) \Biggl[ \sum _{ \vert m \vert = 1}^{\infty} \vert m - \xi \vert ^{p(1 - \lambda_{1}) - 1}a_{m}^{p} \Biggr]^{\frac{1}{p}} \Biggl[ \sum _{ \vert n \vert = 1}^{\infty} \vert n - \eta \vert ^{q(1 - \lambda_{2}) - 1} b_{n}^{q} \Biggr]^{\frac{1}{q}}, \end{aligned} $$

where $2B(\lambda_{1},\lambda_{2})$ ($0 < \lambda_{1},\lambda_{2} \le 1$, $\lambda_{1} + \lambda_{2} = \lambda$, $\xi,\eta\in[0,\frac{1}{2}]$) is the best possible constant factor. In addition, Yang et al. [17, 18] also carried out a few similar works.

In this paper, we present a new discrete Mulholland-type inequality in the whole plane with a best possible constant factor that is similar to that in (4) via introducing multi-parameters, applying weight coefficients, and using Hermite–Hadamard’s inequality. Moreover, the equivalent forms, some particular cases, and the operator expressions are considered.

## An example and two lemmas

In what follows, we assume that $0 < \lambda_{1},\lambda_{2} < 1$, $\lambda_{1} + \lambda_{2} = \lambda\le1$, $\xi,\eta\in[0,\frac{1}{2}]$, $\alpha,\beta\in[\arccos\frac{1}{3},\frac{\pi}{2}] $, and

5 $$ k_{\gamma} (\lambda_{1}): = \frac{2\pi^{2}\csc^{2}\gamma}{ \lambda^{2}\sin^{2}(\frac{\pi\lambda_{1}}{\lambda} )}\quad(\gamma= \alpha,\beta). $$

### Remark 1

In view of the assumptions that $\xi,\eta\in [0,\frac{1}{2}]$, $\alpha,\beta\in[\arccos\frac{1}{3},\frac{\pi}{2}] $, it follows that

$$\biggl(\frac{3}{2} \pm\eta\biggr) (1 \mp\cos\beta) \ge1\quad\mbox{and} \quad\biggl( \frac{3}{2} \pm \xi\biggr) (1 \mp\cos\alpha) \ge1. $$

### Example 1

For $u > 0 $, we set $g(u): = \frac{\ln u}{u - 1}$ ($u > 0$), $g(1): = \lim_{u \to1}g(u) = 1 $. Then we have $g(u) > 0$, $g'(u) < 0$, $g''(u) > 0$ ($u > 0$). In fact, we find

$$g(u) = \frac{\ln[1 + (u - 1)]}{u - 1} = \sum_{k = 0}^{\infty} ( - 1)^{k}\frac{(u - 1)^{k}}{k + 1} = \sum_{k = 0}^{\infty} \frac{( - 1)^{k}k!}{k + 1} \frac{(u - 1)^{k}}{k!}\quad( - 1 < u - 1 \le1), $$

and then $g^{(k)}(1) = \frac{( - 1)^{k}k!}{k + 1}$ ($k = 0,1,2, \ldots$). Hence, $g^{(0)}(1) = g(1)$, $g'(1) = - \frac{1}{2}$, $g''(1) = \frac{2}{3} $. It is evident that $g(u) > 0 $. We obtain $g'(u) = \frac{h(u)}{u(u - 1)^{2}}$, $h(u): = u - 1 - u\ln u $. Since

$$h'(u) = - \ln u > 0\quad(0 < u < 1);\qquad h'(u) < 0\quad(u > 1), $$

it follows that $h_{\max} = h(1) = 0 $ and $h(u) < 0$ ($u \ne1$). Then we have $g'(u) < 0$ ($u \ne1$). In view of $g'(1) = - \frac{1}{2} < 0 $, it follows that $g'(u) < 0$ ($u > 0$). We find

$$g''(u) = \frac{J(u)}{u^{2}(u - 1)^{3}},\qquad J(u): = - (u - 1)^{2} - 2u(u - 1) + 2u^{2}\ln u, $$

$J'(u) = - 4(u - 1) + 4u\ln u $, and

$$J''(u) = 4\ln u < 0 \quad(0 < u < 1);\qquad J''(u) > 0\quad(u > 1). $$

It follows that $J'_{\min} = J'(1) = 0 $, $J'(u) > 0$ ($u \ne1$) and $J(u)$ is strictly increasing. In view of $J(1) = 0 $, we have

$$J(u) < 0\quad(0 < u < 1);\qquad J(u) > 0\quad(u > 1), $$

and $g''(u) > 0$ ($u \ne1$). Since $g''(1) = \frac{2}{3} > 0 $, we find $g''(u) > 0$ ($u > 0$).

For $0 < \lambda\le1$, $0 < \lambda_{2} < 1 $, setting $G(u): = g(u^{\lambda} )u^{\lambda_{2} - 1}$ ($u > 0$), we still have $G(u) > 0 $, $G'(u) = \lambda g'(u^{\lambda} )u^{\lambda+ \lambda_{2} - 2} + (\lambda_{2} - 1)g(u^{\lambda} )u^{\lambda_{2} - 2} < 0 $, and

$$\begin{aligned} G''(u) = {}& \lambda^{2}g''\bigl(u^{\lambda} \bigr)u^{2\lambda+ \lambda_{2} - 3} + \lambda(\lambda+ \lambda_{2} - 2)g'\bigl(u^{\lambda} \bigr)u^{\lambda+ \lambda_{2} - 3} \\ &+ \lambda(\lambda_{2} - 1)g'\bigl(u^{\lambda} \bigr)u^{\lambda+ \lambda_{2} - 3} + (\lambda_{2} - 1) (\lambda_{2} - 2)g\bigl(u^{\lambda} \bigr)u^{\lambda_{2} - 3} > 0. \end{aligned} $$

We set $F(x,y): = \frac{\ln(x/y)}{x^{\lambda} - y^{\lambda}} (\frac{y}{x})^{\lambda_{2} - 1}$ ($x,y > 0$). Since $F(x,y) = \frac{1}{x^{\lambda}} G(\frac{y}{x})$, we have

$$F(x,y) > 0,\qquad\frac{\partial}{\partial y}F(x,y) < 0,\qquad\frac{\partial^{2}}{\partial y^{2}}F(x,y) > 0. $$

Hence, for $x,y > 1 $, we still have

$$\frac{1}{y}F(\ln x,\ln y) > 0,\qquad\frac{\partial}{\partial y}\biggl( \frac {1}{y}F(\ln x,\ln y)\biggr) < 0,\qquad\frac{\partial^{2}}{\partial y^{2}}\biggl( \frac{1}{y}F(\ln x,\ln y)\biggr) > 0. $$

### Lemma 1

(cf. [19])

*If* $f(u) > 0$, $f'(u) < 0$, $f''(u) > 0$ ($u > \frac{3}{2}$) *and* $\int_{\frac{3}{2}}^{\infty} f(u)\,du < \infty $, *then we have the following Hermite–Hadamard’s inequality*:

$$\int_{k}^{k + 1} f(u)\,du < f(k) < \int_{k - \frac{1}{2}}^{k + \frac{1}{2}} f(u)\,du \quad\bigl(k \in\mathbf{N} \setminus\{ 1\} \bigr), $$

*and then*

6 $$ \int_{2}^{\infty} f(u)\,du < \sum _{k = 2}^{\infty} f(k) < \int_{\frac{3}{2}}^{\infty} f(u)\,du. $$

For $|x|,|y| \ge\frac{3}{2} $, let the functions

$$A_{\xi,\alpha} (x): = \vert x - \xi \vert + (x - \xi)\cos\alpha, $$

$A_{\eta,\beta} (y) = |y - \eta| + (y - \eta)\cos\beta $, and

7 $$ k(x,y): = \frac{\ln(\ln A_{\xi,\alpha} (x)/\ln A_{\eta,\beta} (y))}{\ln^{\lambda} A_{\xi,\alpha} (x) - \ln^{\lambda} A_{\eta,\beta} (y)}. $$

We define two weight coefficients as follows:

8 $$\begin{aligned}& \omega(\lambda_{2},m): = \sum_{ \vert n \vert = 2}^{\infty} \frac{k(m,n)}{A_{\eta,\beta} (n)} \cdot \frac{\ln^{\lambda_{1}}A_{\xi,\alpha} (m)}{\ln^{1 - \lambda_{2}}A_{\eta,\beta} (n)}, \quad \vert m \vert \in\mathbf{N} \setminus\{ 1\}, \end{aligned}$$

9 $$\begin{aligned}& \varpi(\lambda_{1},n): = \sum_{ \vert m \vert = 2}^{\infty} \frac{k(m,n)}{A_{\xi,\alpha} (m)} \cdot \frac{\ln^{\lambda_{2}}A_{\eta,\beta} (n)}{\ln^{1 - \lambda_{1}}A_{\xi,\alpha} (m)}, \quad \vert n \vert \in\mathbf{N} \setminus\{ 1\}, \end{aligned}$$

where $\sum_{|j| = 2}^{\infty} \cdots= \sum_{j = - 2}^{ - \infty} \cdots+ \sum_{j = 2}^{\infty} \cdots$ ($j = m,n$).

### Lemma 2

*The inequalities*

10 $$ k_{\beta} (\lambda_{1}) \bigl(1 - \theta( \lambda_{2},m)\bigr) < \omega (\lambda_{2},m) < k_{\beta} (\lambda_{1}), \quad \vert m \vert \in\mathbf{N} \setminus \{ 1\} $$

*are valid*, *where*

11 $$\begin{aligned}[b] \theta(\lambda_{2},m)&: = \biggl[\frac{\lambda}{\pi} \sin\biggl( \frac{\pi \lambda_{1}}{\lambda} \biggr)\biggr]^{2} \int_{0}^{\frac{\ln[(2 + \eta)(1 + \cos \beta )]}{\ln A_{\xi,\alpha} (m)}} \frac{\ln u}{u^{\lambda} - 1} u^{\lambda _{2} - 1} \,du \\&= O\biggl(\frac{1}{\ln^{\lambda_{2}/2}A_{\xi,\alpha} (m)}\biggr) \in (0,1).\end{aligned} $$

### Proof

For $|m| \in\mathbf{N}\setminus\{ 1\} $, let

$$\begin{gathered} k^{(1)}(m,y): = \frac{\ln\ln A_{\xi,\alpha} (m) - \ln \ln [(y - \eta)(\cos\beta- 1)]}{\ln^{\lambda} A_{\xi,\alpha} (m) - \ln^{\lambda} [(y - \eta)(\cos\beta- 1)]},\quad y < - \frac{3}{2}, \\ k^{(2)}(m,y): = \frac{\ln\ln A_{\xi,\alpha} (m) - \ln\ln[(y - \eta )(\cos\beta+ 1)]}{\ln^{\lambda} A_{\xi,\alpha} (m) - \ln^{\lambda} [(y - \eta)(\cos\beta+ 1)]},\quad y > \frac{3}{2}. \end{gathered} $$

Then we have

$$k^{(1)}(m, - y) = \frac{\ln\ln A_{\xi,\alpha} (m) - \ln\ln[(y + \eta)(1 - \cos\beta)]}{\ln^{\lambda} A_{\xi,\alpha} (m) - \ln^{\lambda} [(y + \eta)(1 - \cos\beta)]},\quad y > \frac{3}{2}, $$

yields

12 $$ \begin{aligned}[b] \omega(\lambda_{2},m) = {}&\sum _{n = - 2}^{ - \infty} \frac{k^{(1)}(m,n)\ln^{\lambda_{1}}A_{\xi,\alpha} (m)}{(n - \eta)(\cos \beta- 1)\ln^{1 - \lambda_{2}}[(n - \eta)(\cos\beta- 1)]} \\ &+ \sum_{n = 2}^{\infty} \frac{k^{(2)}(m,n)\ln^{\lambda_{1}}A_{\xi ,\alpha} (m)}{(n - \eta)(1 + \cos\beta)\ln^{1 - \lambda_{2}}[(n - \eta)(1 + \cos \beta)]} \\ ={}& \frac{\ln^{\lambda_{1}}A_{\xi,\alpha} (m)}{1 - \cos\beta} \sum_{n = 2}^{\infty} \frac{k^{(1)}(m, - n)}{(n + \eta)\ln^{1 - \lambda_{2}}[(n + \eta)(1 - \cos\beta)]} \\ &+ \frac{\ln^{\lambda_{1}}A_{\xi,\alpha} (m)}{1 + \cos\beta} \sum_{n = 2}^{\infty} \frac{k^{(2)}(m,n)}{(n - \eta)\ln^{1 - \lambda_{2}}[(n - \eta)(1 + \cos\beta)]}. \end{aligned} $$

In virtue of $0 < \lambda\le1$, $0 < \lambda_{2} < 1 $, and Example 1, we find that for $y > \frac{3}{2} $,

$$\begin{gathered} \frac{k^{(i)}(m,( - 1)^{i}y)}{(y - ( - 1)^{i}\eta)\ln^{1 - \lambda_{2}}[(y - ( - 1)^{i}\eta)(1 + ( - 1)^{i}\cos\beta)]} > 0, \\ \frac{d}{dy}\frac{k^{(i)}(m,( - 1)^{i}y)}{(y - ( - 1)^{i}\eta)\ln^{1 - \lambda_{2}}[(y - ( - 1)^{i}\eta)(1 + ( - 1)^{i}\cos\beta)]} < 0, \\ \frac{d^{2}}{dy^{2}}\frac{k^{(i)}(m,( - 1)^{i}y)}{(y - ( - 1)^{i}\eta )\ln^{1 - \lambda_{2}}[(y - ( - 1)^{i}\eta)(1 + ( - 1)^{i}\cos\beta )]} > 0\quad(i = 1,2), \end{gathered} $$

it follows that

$$\frac{k^{(i)}(m,( - 1)^{i}y)}{(y - ( - 1)^{i}\eta)\ln^{1 - \lambda_{2}}[(y - ( - 1)^{i}\eta)(1 + ( - 1)^{i}\cos\beta)]}\quad(i = 1,2) $$

are strictly decreasing and convex in $( \frac{3}{2},\infty )$. Then, by (5), (12) yields

$$\begin{aligned} \omega(\lambda_{2},m) < {}& \frac{\ln^{\lambda_{1}}A_{\xi,\alpha} (m)}{1 - \cos\beta} \int_{\frac{3}{2}}^{\infty} \frac{k^{(1)}(m, - y)}{(y + \eta)\ln^{1 - \lambda_{2}}[(y + \eta)(1 - \cos\beta)]} \,dy \\ &+ \frac{\ln^{\lambda_{1}}A_{\xi,\alpha} (m)}{1 + \cos\beta} \int_{\frac{3}{2}}^{\infty} \frac{k^{(2)}(m,y)}{(y - \eta)\ln^{1 - \lambda_{2}}[(y - \eta)(1 + \cos\beta)]} \,dy. \end{aligned} $$

Setting $u = \frac{\ln[(y + \eta)(1 - \cos\beta)]}{\ln A_{\xi,\alpha} (m)}$ ($u = \frac{\ln[(y - \eta)(1 + \cos\beta)]}{\ln A_{\xi,\alpha} (m)}$) in the above first (second) integral, in view of Remark 1, we obtain

$$\begin{aligned} \omega(\lambda_{2},m) &< \biggl( \frac{1}{1 - \cos\beta} + \frac{1}{1 + \cos\beta} \biggr) \int_{0}^{\infty} \frac{\ln u}{u^{\lambda} - 1} u^{\lambda_{2} - 1} \,du \\ &= \frac{2\csc^{2}\beta}{\lambda^{2}} \int_{0}^{\infty} \frac{\ln v}{v - 1} v^{(\lambda_{2}/\lambda) - 1}\,dv = \frac{2\pi^{2}\csc^{2}\beta}{ \lambda^{2}\sin^{2}(\frac{\pi\lambda_{1}}{\lambda} )} = k_{\beta} (\lambda_{1}) \end{aligned} $$

by simplifications. Similarly, by (5), (12) also yields

$$\begin{aligned} \omega(\lambda_{2},m) >{}& \frac{\ln^{\lambda_{1}}A_{\xi,\alpha} (m)}{1 - \cos\beta} \int_{2}^{\infty} \frac{k^{(1)}(m, - y)}{(y + \eta)\ln^{1 - \lambda_{2}}[(y + \eta)(1 - \cos\beta)]} \,dy \\ &+ \frac{\ln^{\lambda_{1}}A_{\xi,\alpha} (m)}{1 + \cos\beta} \int_{2}^{\infty} \frac{k^{(2)}(m,y)}{(y - \eta)\ln^{1 - \lambda _{2}}[(y - \eta)(1 + \cos\beta)]} \,dy \\ \ge{}& \biggl(\frac{1}{1 - \cos\beta} + \frac{1}{1 + \cos\beta} \biggr) \int_{\frac{\ln[(2 + \eta)(1 + \cos\beta)]}{\ln A_{\xi,\alpha} (m)}}^{\infty} \frac{\ln u}{u^{\lambda} - 1} u^{\lambda_{2} - 1} \,du \\ ={}& k_{\beta} (\lambda_{1}) - 2\csc^{2}\beta \int_{0}^{\frac{\ln[(2 + \eta )(1 + \cos\beta)]}{\ln A_{\xi,\alpha} (m)}} \frac{\ln u}{u^{\lambda} - 1}u^{\lambda_{2} - 1} \,du \\ ={}& k_{\beta} (\lambda_{1}) \bigl(1 - \theta( \lambda_{2},m)\bigr) > 0, \end{aligned} $$

where $\theta(\lambda_{2},m)$ (<1) is indicated by (11). Since

$$\frac{\ln u}{u^{\lambda} - 1}u^{\lambda_{2}/2} \to0\quad\bigl(u \to0^{ +} \bigr);\qquad \frac{\ln u}{u^{\lambda} - 1}u^{\lambda_{2}/2} \to\frac{1}{\lambda} \quad(u \to1), $$

there exists a positive constant *C* such that $\frac{\ln u}{u^{\lambda} - 1}u^{\lambda_{2}/2} \le C$ ($0 < u \le1$), and then for $A_{\xi,\alpha} (m) \ge(2 + \eta)(1 + \cos\beta)$, we have

13 $$ \begin{aligned}[b] 0 &< \theta(\lambda_{2},m) \le C\biggl[ \frac{\lambda}{\pi} \sin\biggl(\frac{\pi \lambda_{1}}{\lambda} \biggr)\biggr]^{2} \int_{0}^{\frac{\ln[(2 + \eta)(1 + \cos \beta )]}{\ln A_{\xi,\alpha} (m)}} u^{\frac{\lambda_{2}}{2} - 1} \,du \\ &= \frac{2C}{\lambda_{2}}\biggl[\frac{\lambda}{\pi} \sin\biggl(\frac{\pi \lambda_{1}}{\lambda} \biggr)\biggr]^{2}\biggl\{ \frac{\ln[(2 + \eta)(1 + \cos\beta )]}{\ln A_{\xi,\alpha} (m)}\biggr\} ^{\frac{\lambda_{2}}{2}}. \end{aligned} $$

Hence, (10) and (11) are valid. □

Similarly, we have the following.

### Lemma 3

*For* $0 < \lambda\le1$, $0 < \lambda_{1} < 1 $, *the inequalities*

14 $$ k_{\alpha} (\lambda_{1}) \bigl(1 - \tilde{\theta} ( \lambda_{1},n)\bigr) < \varpi (\lambda_{1},n) < k_{\alpha} (\lambda_{1}), \quad \vert n \vert \in\mathbf{N} \setminus \{ 1\} $$

*are valid*, *where*

15 $$\begin{aligned}[b] \tilde{\theta} (\lambda_{1},n)&: = \biggl[\frac{\lambda}{\pi} \sin \biggl(\frac{\pi \lambda_{1}}{\lambda} \biggr)\biggr]^{2} \int_{0}^{\frac{\ln[(2 + \xi)(1 + \cos \alpha )]}{\ln A_{\eta,\beta} (n)}} \frac{\ln u}{u^{\lambda} - 1} u^{\lambda _{1} - 1} \,du\\& = O\biggl(\frac{1}{\ln^{\lambda_{1}/2}A_{\eta,\beta} (n)}\biggr) \in (0,1).\end{aligned} $$

### Lemma 4

*If* $(\varsigma,\gamma) = (\xi,\alpha )$ (*or* $(\eta,\beta )$), $\rho> 0 $, *then we have*

16 $$ H_{\rho} (\varsigma,\gamma): = \sum_{|k| = 2}^{\infty} \frac{\ln^{ - 1 - \rho} A_{\varsigma,\gamma} (k)}{A_{\varsigma,\gamma} (k)} = \frac {1}{\rho} \bigl(2\csc^{2}\gamma+ o(1) \bigr) \quad\bigl(\rho\to0^{ +} \bigr). $$

### Proof

According to (5), we obtain

$$\begin{aligned} H_{\rho} (\varsigma,\gamma) &= \sum _{k = - 2}^{ - \infty} \frac{\ln^{ - 1 - \rho} [(k - \varsigma)(\cos\gamma- 1)]}{(k - \varsigma )(\cos\gamma- 1)} + \sum _{k = 2}^{\infty} \frac{\ln^{ - 1 - \rho} [(k - \varsigma)(\cos\gamma+ 1)]}{(k - \varsigma)(\cos\gamma+ 1)} \\ &= \sum_{k = 2}^{\infty} \biggl\{ \frac{\ln^{ - 1 - \rho} [(k + \varsigma)(1 - \cos\gamma)]}{(k - \varsigma)(1 - \cos\gamma)} + \frac{\ln^{ - 1 - \rho} [(k - \varsigma)(\cos\gamma+ 1)]}{(k - \varsigma)(\cos\gamma+ 1)}\biggr\} \\ &< \int_{\frac{3}{2}}^{\infty} \biggl\{ \frac{\ln^{ - 1 - \rho} [(y + \varsigma )(1 - \cos\gamma)]}{(y - \varsigma)(1 - \cos\gamma)} + \frac{\ln^{ - 1 - \rho} [(y - \varsigma)(\cos\gamma+ 1)]}{(y - \varsigma)(\cos \gamma+ 1)}\biggr\} \,dy \\ & = \frac{1}{\rho} \biggl\{ \frac{\ln^{ - \rho} [(\frac{3}{2} + \varsigma)(1 - \cos\gamma)]}{1 - \cos\gamma} + \frac{\ln^{ - \rho} [(\frac{3}{2} - \varsigma)(1 + \cos\gamma)]}{1 + \cos\gamma} \biggr\} \\ &= \frac{1}{\rho} \biggl(\frac{1}{1 - \cos\gamma} + \frac{1}{1 + \cos\gamma } + o_{1}(1)\biggr) = \frac{1}{\rho} \bigl(2\csc^{2} \gamma+ o_{1}(1)\bigr)\quad \bigl(\rho\to0^{ +} \bigr), \end{aligned} $$

and

$$\begin{aligned} H_{\rho} (\varsigma,\gamma) &= \sum _{k = 2}^{\infty} \biggl\{ \frac{\ln^{ - 1 - \rho} [(k + \varsigma)(1 - \cos\gamma)]}{(k - \varsigma)(1 - \cos\gamma)} + \frac{\ln^{ - 1 - \rho} [(k - \varsigma )(\cos\gamma+ 1)]}{(k - \varsigma)(\cos\gamma+ 1)}\biggr\} \\ &> \int_{2}^{\infty} \biggl\{ \frac{\ln^{ - 1 - \rho} [(y + \varsigma)(1 - \cos \gamma)]}{(y - \varsigma)(1 - \cos\gamma)} + \frac{\ln^{ - 1 - \rho} [(y - \varsigma)(\cos\gamma+ 1)]}{(y - \varsigma)(\cos\gamma+ 1)}\biggr\} \,dy \\ &= \frac{1}{\rho} \biggl\{ \frac{\ln^{ - \rho} [(2 + \varsigma )(1 - \cos\gamma)]}{1 - \cos\gamma} + \frac{\ln^{ - \rho} [(2 - \varsigma)(1 + \cos\gamma)]}{1 + \cos\gamma} \biggr\} \\ &= \frac{1}{\rho} \biggl(\frac{1}{1 - \cos\gamma} + \frac{1}{1 + \cos\gamma } + o_{2}(1)\biggr) = \frac{1}{\rho} \bigl(2\csc^{2}\gamma+ o_{2}(1)\bigr) \quad\bigl(\rho\to0^{ +} \bigr). \end{aligned} $$

Therefore, (16) is valid. □

## Main results

### Theorem 1

*Suppose that* $p > 1$, $\frac{1}{p} + \frac{1}{q} = 1 $, *we set*

17 $$ k(\lambda_{1}): = k_{\beta}^{1/p}( \lambda_{1})k_{\alpha}^{1/q}(\lambda_{1}) = \frac{2\pi^{2}\csc^{2/p}\beta\csc^{2/q}\alpha}{[\lambda\sin(\frac {\pi \lambda_{1}}{\lambda} )]^{2}}. $$

*If* $a_{m},b_{n} \ge0$ ($|m|,|n| \in\mathbf{N}\setminus\{ 1\} $) *satisfy*

$$0 < \sum_{|m| = 2}^{\infty} \frac{\ln^{p(1 - \lambda_{1}) - 1}A_{\xi,\alpha} (m)}{(A_{\xi,\alpha} (m))^{1 - p}} a_{m}^{p} < \infty ,\qquad0 < \sum_{|n| = 2}^{\infty} \frac{\ln^{q(1 - \lambda_{2}) - 1}A_{\eta,\beta} (n)}{(A_{\eta,\beta} (n))^{1 - q}} b_{n}^{q} < \infty, $$

*then we obtain the following equivalent inequalities*:

18 $$\begin{aligned}& \begin{aligned}[b] I&: = \sum_{|n| = 2}^{\infty} \sum_{|m| = 2}^{\infty} \frac{\ln(\ln A_{\xi,\alpha} (m)/\ln A_{\eta,\beta} (n))}{\ln^{\lambda} A_{\xi,\alpha} (m) - \ln^{\lambda} A_{\eta,\beta} (n)} a_{m}b_{n} \\ &< \frac{2\pi^{2}\csc^{2/p}\beta\csc^{2/q}\alpha}{[\lambda\sin(\frac {\pi \lambda_{1}}{\lambda} )]^{2}} \Biggl[ \sum_{|m| = 2}^{\infty} \frac{\ln^{p(1 - \lambda_{1}) - 1}A_{\xi,\alpha} (m)}{(A_{\xi,\alpha} (m))^{1 - p}}a_{m}^{p} \Biggr]^{\frac{1}{p}}\\ &\quad{}\times \Biggl[ \sum_{|n| = 2}^{\infty} \frac{\ln^{q(1 - \lambda_{2}) - 1}A_{\eta,\beta} (n)}{(A_{\eta,\beta} (n))^{1 - q}}b_{n}^{q} \Biggr]^{\frac{1}{q}}, \end{aligned} \end{aligned}$$

19 $$\begin{aligned}& \begin{aligned}[b] J&: = \Biggl\{ \sum_{|n| = 2}^{\infty} \frac{\ln^{p\lambda_{2} - 1}A_{\eta,\beta} (n)}{A_{\eta,\beta} (n)} \Biggl[ \sum_{|m| = 2}^{\infty} \frac{\ln(\ln A_{\xi,\alpha} (m)/\ln A_{\eta,\beta} (n))}{\ln^{\lambda} A_{\xi,\alpha} (m) - \ln^{\lambda} A_{\eta,\beta} (n)}a_{m} \Biggr]^{p} \Biggr\} ^{\frac{1}{p}} \\ &< \frac{2\pi^{2}\csc^{2/p}\beta\csc^{2/q}\alpha}{[\lambda\sin(\frac {\pi \lambda_{1}}{\lambda} )]^{2}} \Biggl[ \sum_{|m| = 2}^{\infty} \frac{\ln^{p(1 - \lambda_{1}) - 1}A_{\xi,\alpha} (m)}{(A_{\xi,\alpha} (m))^{1 - p}}a_{m}^{p} \Biggr]^{\frac{1}{p}}. \end{aligned} \end{aligned}$$

*Particularly*, (i) *for* $\alpha= \beta= \frac{\pi}{2}$, $\xi,\eta\in [0,\frac{1}{2}] $, *we have the following equivalent inequalities*:

20 $$\begin{aligned}& \sum_{ \vert n \vert = 2}^{\infty} \sum _{ \vert m \vert = 2}^{\infty} \frac{\ln( \vert m - \xi \vert / \vert n - \eta \vert )a_{m}b_{n}}{\ln^{\lambda} \vert m - \xi \vert - \ln^{\lambda} \vert n - \eta \vert } \\& \quad< \frac{2\pi^{2}}{[\lambda\sin(\frac{\pi\lambda_{1}}{\lambda} )]^{2}} \Biggl[ \sum_{ \vert m \vert = 2}^{\infty} \frac{\ln^{p(1 - \lambda_{1}) - 1} \vert m - \xi \vert }{ \vert m - \xi \vert ^{1 - p}}a_{m}^{p} \Biggr]^{\frac{1}{p}} \Biggl[ \sum_{ \vert n \vert = 2}^{\infty} \frac{\ln^{q(1 - \lambda_{2}) - 1} \vert n - \eta \vert }{ \vert n - \eta \vert ^{1 - q}}b_{n}^{q} \Biggr]^{\frac{1}{q}}, \end{aligned}$$

21 $$\begin{aligned}& \Biggl[ \sum_{ \vert n \vert = 2}^{\infty} \frac{\ln^{p\lambda_{2} - 1} \vert n - \eta \vert }{ \vert n - \eta \vert } \Biggl( \sum_{ \vert m \vert = 2}^{\infty} \frac{\ln( \vert m - \xi \vert / \vert n - \eta \vert )a_{m}}{\ln^{\lambda} \vert m - \xi \vert - \ln^{\lambda} \vert n - \eta \vert } \Biggr)^{p} \Biggr]^{\frac{1}{p}} \\& \quad< \frac{2\pi^{2}}{[\lambda\sin(\frac{\pi \lambda_{1}}{\lambda} )]^{2}} \Biggl[ \sum _{ \vert m \vert = 2}^{\infty} \frac{\ln^{p(1 - \lambda_{1}) - 1} \vert m - \xi \vert }{ \vert m - \xi \vert ^{1 - p}}a_{m}^{p} \Biggr]^{\frac{1}{p}}. \end{aligned}$$

(ii) *For* $\xi= \eta= 0$, $\alpha,\beta\in[\arccos\frac{1}{3},\frac{\pi}{ 2}] $, *we have the following equivalent inequalities*:

22 $$\begin{aligned}& \begin{aligned}[b] &\sum_{ \vert n \vert = 2}^{\infty} \sum_{ \vert m \vert = 2}^{\infty} \frac{\ln[\ln( \vert m \vert + m\cos \alpha)/\ln( \vert n \vert + n\cos\beta)]}{\ln^{\lambda} ( \vert m \vert + m\cos\alpha) - \ln^{\lambda} ( \vert n \vert + n\cos\beta)} a_{m}b_{n} \\ &\quad< \frac{2\pi^{2}\csc^{2/p}\beta\csc^{2/q}\alpha}{[\lambda\sin (\frac{\pi \lambda_{1}}{\lambda} )]^{2}}\\&\qquad{}\times \Biggl[ \sum_{ \vert m \vert = 2}^{\infty} \frac{\ln^{p(1 - \lambda_{1}) - 1}( \vert m \vert + m\cos\alpha)}{( \vert m \vert + m\cos\alpha)^{1 - p}}a_{m}^{p} \Biggr]^{\frac{1}{p}} \Biggl[ \sum_{ \vert n \vert = 2}^{\infty} \frac{\ln^{q(1 - \lambda_{2}) - 1}( \vert n \vert + n\cos\beta)}{( \vert n \vert + n\cos \beta )^{1 - q}}b_{n}^{q} \Biggr]^{\frac{1}{q}}, \end{aligned} \end{aligned}$$

23 $$\begin{aligned}& \begin{aligned}[b]& \Biggl\{ \sum_{ \vert n \vert = 2}^{\infty} \frac{\ln^{p\lambda _{2} - 1}( \vert n \vert + n\cos \beta)}{ \vert n \vert + n\cos\beta} \Biggl[ \sum_{ \vert m \vert = 2}^{\infty} \frac{\ln [\ln ( \vert m \vert + m\cos\alpha)/\ln( \vert n \vert + n\cos\beta)]}{\ln^{\lambda} ( \vert m \vert + m\cos \alpha) - \ln^{\lambda} ( \vert n \vert + n\cos\beta)}a_{m} \Biggr]^{p} \Biggr\} ^{\frac{1}{p}} \\ &\quad< \frac{2\pi^{2}\csc^{2/p}\beta\csc^{2/q}\alpha}{[\lambda\sin (\frac{\pi \lambda_{1}}{\lambda} )]^{2}} \Biggl[ \sum_{ \vert m \vert = 2}^{\infty} \frac{\ln^{p(1 - \lambda_{1}) - 1}( \vert m \vert + m\cos\alpha)}{( \vert m \vert + m\cos\alpha)^{1 - p}}a_{m}^{p} \Biggr]^{\frac{1}{p}}. \end{aligned} \end{aligned}$$

### Proof

According to Hölder’s inequality with weight (cf. [20]) and (9), we find

$$\begin{gathered} \Biggl( \sum_{|m| = 2}^{\infty} k(m,n)a_{m} \Biggr)^{p} \\ \quad= \Biggl\{ \sum_{|m| = 2}^{\infty} k(m,n) \biggl[ \frac{(A_{\xi,\alpha} (m))^{\frac{1}{q}}\ln^{\frac{1 - \lambda_{1}}{q}}A_{\xi,\alpha} (m)}{\ln^{\frac{1 - \lambda_{2}}{p}}A_{\eta,\beta} (n)}a_{m} \biggr] \biggl[ \frac{\ln^{\frac{1 - \lambda_{2}}{p}}A_{\eta,\beta} (n)}{(A_{\xi,\alpha} (m))^{\frac{1}{q}}\ln^{\frac{1 - \lambda_{1}}{q}}A_{\xi,\alpha} (m)} \biggr] \Biggr\} ^{p} \\ \quad\le\sum_{|m| = 2}^{\infty} k(m,n) \frac{(A_{\xi,\alpha} (m))^{\frac{p}{q}}\ln^{\frac{(1 - \lambda_{1})p}{q}}A_{\xi,\alpha} (m)}{\ln^{1 - \lambda_{2}}A_{\eta,\beta} (n)}\\ \quad\quad{}\times a_{m}^{p} \Biggl[ \sum _{|m| = 2}^{\infty} k(m,n)\frac{\ln^{\frac{(1 - \lambda_{2})q}{p}}A_{\eta,\beta} (n)}{A_{\xi,\alpha} (m)\ln^{1 - \lambda_{1}}A_{\xi,\alpha} (m)} \Biggr]^{p - 1} \\ \quad= \frac{(\varpi(\lambda_{1},n))^{p - 1}A_{\eta,\beta} (n)}{\ln^{p\lambda_{2} - 1}A_{\eta,\beta} (n)}\sum_{|m| = 2}^{\infty} k(m,n)\frac{(A_{\xi,\alpha} (m))^{\frac{p}{q}}\ln^{\frac{(1 - \lambda_{1})p}{q}}A_{\xi,\alpha} (m)}{A_{\eta,\beta} (n)\ln^{1 - \lambda_{2}}A_{\eta,\beta} (n)}a_{m}^{p}. \end{gathered} $$

Then, by (14), it yields

24 $$ \begin{aligned}[b] J &< k_{\alpha}^{1/q}( \lambda_{1}) \Biggl[ \sum_{|n| = 2}^{\infty} \sum_{|m| = 2}^{\infty} k(m,n)\frac{(A_{\xi,\alpha} (m))^{\frac{p}{q}}\ln^{\frac{(1 - \lambda_{1})p}{q}}A_{\xi,\alpha} (m)}{A_{\eta,\beta} (n)\ln^{1 - \lambda_{2}}A_{\eta,\beta} (n)}a_{m}^{p} \Biggr]^{\frac{1}{p}} \\ &= k_{\alpha}^{1/q}(\lambda_{1}) \Biggl[ \sum _{|m| = 2}^{\infty} \sum _{|n| = 2}^{\infty} k(m,n)\frac{(A_{\xi,\alpha} (m))^{\frac{p}{q}}\ln^{\frac{(1 - \lambda_{1})p}{q}}A_{\xi,\alpha} (m)}{A_{\eta,\beta} (n)\ln^{1 - \lambda_{2}}A_{\eta,\beta} (n)}a_{m}^{p} \Biggr]^{\frac{1}{p}} \\ &= k_{\alpha}^{1/q}(\lambda_{1}) \Biggl[ \sum _{|m| = 2}^{\infty} \omega (\lambda_{2},m) \frac{n^{p(1 - \lambda_{1}) - 1}A_{\xi,\alpha} (m)}{(A_{\xi,\alpha} (m))^{1 - p}}a_{m}^{p} \Biggr]^{\frac{1}{p}}. \end{aligned} $$

Combining (10) and (17), we obtain (19).

Using Hölder’s inequality again, we obtain

25 $$ \begin{aligned}[b] I &= \sum_{|n| = 2}^{\infty} \Biggl[ \frac{(A_{\eta,\beta} (n))^{\frac{ - 1}{p}}}{\ln^{\frac{1}{p} - \lambda_{2}}A_{\eta,\beta} (n)}\sum_{|m| = 2}^{\infty} k(m,n)a_{m} \Biggr] \biggl[ \frac{\ln^{\frac{1}{p} - \lambda_{2}}A_{\eta,\beta} (n)}{(A_{\eta,\beta} (n))^{\frac{ - 1}{p}}}b_{n} \biggr] \\ &\le J \Biggl[ \sum_{|n| = 2}^{\infty} \frac{\ln^{q(1 - \lambda_{2}) - 1}A_{\eta,\beta} (n)}{(A_{\eta,\beta} (n))^{1 - q}} b_{n}^{q} \Biggr]^{\frac{1}{q}}. \end{aligned} $$

Then, according to (19), we obtain (18).

On the other hand, assuming that (18) is valid, we let

$$b_{n}: = \frac{\ln^{p\lambda_{2} - 1}A_{\eta,\beta} (n)}{A_{\eta,\beta} (n)} \Biggl( \sum _{ \vert m \vert = 2}^{\infty} k(m,n)a_{m} \Biggr)^{p - 1},\quad \vert n \vert \in \mathbf{N}\setminus\{ 1\}. $$

According to (24), it follows that $J < \infty $. If $J = 0 $, then (20) is trivially valid; if $J > 0 $, then we have

$$\begin{gathered} \begin{aligned} 0 &< \sum _{|n| = 2}^{\infty} \frac{\ln^{q(1 - \lambda_{2}) - 1}A_{\eta,\beta} (n)}{(A_{\eta,\beta} (n))^{1 - q}} b_{n}^{q} \\&= J^{p} = I \\ &< k(\lambda_{1}) \Biggl[ \sum_{|m| = 2}^{\infty} \frac{\ln^{p(1 - \lambda_{1}) - 1}A_{\xi,\alpha} (m)}{(A_{\xi,\alpha} (m))^{1 - p}}a_{m}^{p} \Biggr]^{\frac{1}{p}} \Biggl[ \sum_{|n| = 2}^{\infty} \frac{\ln^{q(1 - \lambda_{2}) - 1}A_{\eta,\beta} (n)}{(A_{\eta,\beta} (n))^{1 - q}}b_{n}^{q} \Biggr]^{\frac{1}{q}}, \end{aligned} \\ \begin{aligned}J &= \Biggl[ \sum_{|n| = 2}^{\infty} \frac{\ln^{q(1 - \lambda_{2}) - 1}A_{\eta,\beta} (n)}{(A_{\eta,\beta} (n))^{1 - q}} b_{n}^{q} \Biggr]^{\frac{1}{p}} \\&< k( \lambda_{1}) \Biggl[ \sum_{|m| = 2}^{\infty} \frac{\ln^{p(1 - \lambda_{1}) - 1}A_{\xi,\alpha} (m)}{(A_{\xi,\alpha} (m))^{1 - p}}a_{m}^{p} \Biggr]^{\frac{1}{p}}.\end{aligned} \end{gathered} $$

Thus (19) is valid, which is equivalent to (18). □

### Theorem 2

*With regards to the assumptions in Theorem *1, $k(\lambda _{1})$ *is the best possible constant factor in* (18) *and* (19).

### Proof

For $0 < \varepsilon< \min\{ q(1 - \lambda_{1}),q\lambda_{2}\} $, we let $\tilde{\lambda}_{1} = \lambda_{1} + \frac{\varepsilon}{q}$ ($\in(0,1)$), $\tilde{\lambda}_{2} = \lambda_{2} - \frac{\varepsilon}{q}$ ($\in(0,1)$), and

$$\begin{gathered}\tilde{a}_{m}: = \frac{\ln^{\lambda_{1} - \frac{\varepsilon}{p} - 1}A_{\xi,\alpha} (m)}{A_{\xi,\alpha} (m)} = \frac{\ln^{\tilde{\lambda}_{1} - \varepsilon- 1}A_{\xi,\alpha} (m)}{A_{\xi,\alpha} (m)}\quad\bigl( \vert m \vert \in\mathbf{N}\setminus\{ 1\} \bigr), \\ \tilde{b}_{n}: = \frac{\ln^{\lambda_{2} - \frac{\varepsilon}{q} - 1}A_{\eta,\beta} (n)}{A_{\eta,\beta} (n)} = \frac{\ln^{\tilde{\lambda}_{2} - 1}A_{\eta,\beta} (n)}{A_{\eta,\beta} (n)}\quad\bigl( \vert n \vert \in \mathbf{N}\setminus\{ 1\} \bigr). \end{gathered} $$

Then (16) and (14) yield

$$\begin{gathered} \begin{aligned} \tilde{I}_{1}&: = \Biggl[ \sum_{|m| = 2}^{\infty} \frac{\ln^{p(1 - \lambda_{1}) - 1}A_{\xi,\alpha} (m)}{(A_{\xi,\alpha} (m))^{1 - p}}\tilde{a}_{m}^{p} \Biggr]^{\frac{1}{p}} \Biggl[ \sum_{|n| = 2}^{\infty} \frac{\ln^{q(1 - \lambda_{2}) - 1}A_{\eta,\beta} (n)}{(A_{\eta,\beta} (n))^{1 - q}} \tilde{b}_{n}^{q} \Biggr]^{\frac{1}{q}} \\ &= \Biggl[ \sum_{|m| = 2}^{\infty} \frac{\ln^{ - 1 - \varepsilon} A_{\xi,\alpha} (m)}{A_{\xi,\alpha} (m)} \Biggr]^{\frac{1}{p}} \Biggl[ \sum _{|n| = 2}^{\infty} \frac{\ln^{ - 1 - \varepsilon} A_{\eta,\beta} (n)}{A_{\eta,\beta} (n)} \Biggr]^{\frac{1}{q}} \\ &= \frac{1}{\varepsilon} \bigl(2\csc^{2}\alpha+ o(1) \bigr)^{\frac{1}{p}}\bigl(2\csc^{2}\beta+ \tilde{o}(1) \bigr)^{\frac{1}{q}}\quad\bigl(\varepsilon\to0^{ +} \bigr), \end{aligned} \\ \begin{aligned} \tilde{I}&: = \sum_{|n| = 2}^{\infty} \sum_{|m| = 2}^{\infty} k(m,n) \tilde{a}_{m} \tilde{b}_{n} = \sum_{|m| = 2}^{\infty} \sum_{|n| = 2}^{\infty} k(m,n) \frac{\ln^{\tilde{\lambda}_{1} - \varepsilon - 1}A_{\xi,\alpha} (m)}{A_{\xi,\alpha} (m)} \frac{\ln^{\tilde{\lambda}_{2} - 1}A_{\eta,\beta} (n)}{A_{\eta,\beta} (n)} \\ &= \sum_{|m| = 2}^{\infty} \omega(\tilde{ \lambda}_{2},m)\frac{\ln^{ - \varepsilon- 1}A_{\xi,\alpha} (m)}{A_{\xi,\alpha} (m)} > k_{\beta} (\tilde{ \lambda}_{1})\sum_{|m| = 2}^{\infty} \bigl(1 - \theta (\tilde{\lambda}_{2},m)\bigr)\frac{\ln^{ - \varepsilon- 1}A_{\xi,\alpha} (m)}{A_{\xi,\alpha} (m)} \\ &= k_{\beta} (\tilde{\lambda}_{1}) \Biggl[ \sum _{|m| = 2}^{\infty} \frac {\ln^{ - \varepsilon- 1}A_{\xi,\alpha} (m)}{A_{\xi,\alpha} (m)} - \sum _{|m| = 2}^{\infty} \frac{O(\ln^{ - (\frac{\varepsilon}{p} + \frac{\lambda_{2}}{2}) - 1}A_{\xi,\alpha} (m))}{A_{\xi,\alpha} (m)} \Biggr] \\ &= \frac{1}{\varepsilon} k_{\beta} (\tilde{\lambda}_{1}) \quad\bigl(2\csc ^{2}\alpha+ o(1) - \varepsilon O(1)\bigr). \end{aligned} \end{gathered} $$

If there exists a positive number $K \le k(\lambda_{1})$ such that (18) is still valid when replacing $k(\lambda_{1})$ by *K*, then we obtain

$$\varepsilon\tilde{I} = \varepsilon\sum_{|n| = 2}^{\infty} \sum_{|m| = 2}^{\infty} k(m,n) \tilde{a}_{m} \tilde{b}_{n} < \varepsilon K\tilde{I}_{1}. $$

Hence, in view of the above results, it follows that

$$k_{\beta} \biggl(\lambda_{1} + \frac{\varepsilon}{q}\biggr) \bigl(2\csc^{2}\alpha+ o(1) - \varepsilon O(1)\bigr) < K\bigl(2 \csc^{2}\alpha+ o(1)\bigr)^{\frac{1}{p}}\bigl(2\csc ^{2} \beta + \tilde{o}(1)\bigr)^{\frac{1}{q}}, $$

and then

$$\frac{4\pi^{2}}{[\lambda\sin(\frac{\pi\lambda_{1}}{\lambda} )]^{2}}\csc^{2}\beta\csc^{2}\alpha\le2K \csc^{\frac{2}{p}}\alpha \csc^{\frac{2}{q}}\beta\quad\bigl(\varepsilon \to0^{ +} \bigr), $$

namely

$$k(\lambda_{1}) = \frac{2\pi^{2}}{[\lambda\sin(\frac{\pi \lambda_{1}}{\lambda} )]^{2}}\csc^{\frac{2}{p}}\beta \csc^{\frac{2}{q}}\alpha\le K. $$

Hence, $K = k(\lambda_{1})$ is the best possible constant factor in (18).

$k(\lambda_{1})$ in (19) is still the best possible constant factor. Otherwise we would reach a contradiction by (25) that $k(\lambda_{1})$ in (18) is not the best possible constant factor. □

## Operator expressions and a remark

Let $\varphi(m): = \frac{\ln^{p(1 - \lambda_{1}) - 1}A_{\xi,\alpha} (m)}{(A_{\xi,\alpha} (m))^{1 - p}}$ ($|m| \in\mathbf{N}\setminus\{ 1\} $), and $\psi(n): = \frac{\ln^{q(1 - \lambda_{2}) - 1}A_{\eta,\beta} (n)}{(A_{\eta,\beta} (n))^{1 - q}} $, wherefrom

$$\psi^{1 - p}(n): = \frac{\ln^{p\lambda_{2} - 1}A_{\eta,\beta} (n)}{A_{\eta,\beta} (n)}\quad\bigl( \vert n \vert \in \mathbf{N}\setminus\{ 1\} \bigr). $$

We define the real weighted normed function spaces as follows:

$$\begin{gathered} l_{p,\varphi}: = \Biggl\{ a = \{ a_{m}\}_{ \vert m \vert = 2}^{\infty}; \Vert a \Vert _{p,\varphi} = \Biggl( \sum_{ \vert m \vert = 2}^{\infty} \varphi (m) \vert a_{m} \vert ^{p} \Biggr)^{\frac{1}{p}} < \infty \Biggr\} , \\ l_{q,\psi}: = \Biggl\{ b = \{ b_{n}\}_{ \vert n \vert = 2}^{\infty}; \Vert b \Vert _{q,\psi} = \Biggl( \sum_{ \vert n \vert = 2}^{\infty} \psi(n) \vert b_{n} \vert ^{q} \Biggr)^{\frac {1}{q}} < \infty \Biggr\} , \\ l_{p,\psi^{1 - p}}: = \Biggl\{ c = \{ c_{n}\}_{ \vert n \vert = 2}^{\infty}; \Vert c \Vert _{p,\psi^{1 - p}} = \Biggl( \sum_{ \vert n \vert = 2}^{\infty} \psi^{1 - p}(n) \vert c_{n} \vert ^{p} \Biggr)^{\frac{1}{p}} < \infty \Biggr\} . \end{gathered} $$

For $a = \{ a_{m}\}_{|m| = 2}^{\infty} \in l_{p,\varphi} $, we let $c_{n} = \sum_{|m| = 2}^{\infty} k(m,n)a_{m} $ and $c = \{ c_{n}\}_{|n| = 2}^{\infty} $, it follows by (19) that $\|c\|_{p,\psi^{1 - p}} < k(\lambda_{1})\|a\|_{p,\varphi} $, namely $c \in l_{p,\psi^{1 - p}} $.

Further, we define a Mulholland-type operator $T:l_{p,\varphi} \to l_{p,\psi^{1 - p}} $ as follows: For $a_{m} \ge0$, $a = \{ a_{m}\}_{|m| = 2}^{\infty} \in l_{p,\varphi} $, there exists a unique representation $Ta = c \in l_{p,\psi^{1 - p}} $. We also define the following formal inner product of *Ta* and $b = \{ b_{n}\}_{|n| = 2}^{\infty} \in l_{q,\psi}$ ($b_{n} \ge 0$):

26 $$ (Ta,b): = \sum_{|n| = 2}^{\infty} \sum _{|m| = 2}^{\infty} k(m,n) a_{m}b_{n}. $$

Hence, we can respectively rewrite (18) and (19) as the following operator expressions:

27 $$\begin{aligned}& (Ta,b) < k(\lambda_{1}) \Vert a \Vert _{p,\varphi} \Vert b \Vert _{q,\psi}, \end{aligned}$$

28 $$\begin{aligned}& \Vert Ta \Vert _{p,\psi^{1 - p}} < k(\lambda_{1}) \Vert a \Vert _{p,\varphi}. \end{aligned}$$

It follows that the operator *T* is bounded with

29 $$ \Vert T \Vert : = \sup_{a( \ne\theta) \in l_{p,\varphi}} \frac{ \Vert Ta \Vert _{p,\psi ^{1 - p}}}{ \Vert a \Vert _{p,\varphi}} \le k( \lambda_{1}). $$

Since $k(\lambda_{1})$ in (19) is the best possible constant factor, we obtain

30 $$ \Vert T \Vert = k(\lambda_{1}) = \frac{2\pi^{2}\csc^{2/p}\beta\csc^{2/q}\alpha}{ [\lambda\sin(\frac{\pi\lambda_{1}}{\lambda} )]^{2}}. $$

### Remark 2

(i) For $\xi= \eta= 0 $ in (20), we have the following new inequality:

31 $$\begin{aligned}[b] &\sum_{ \vert n \vert = 2}^{\infty} \sum _{ \vert m \vert = 2}^{\infty} \frac{\ln(\ln \vert m \vert /\ln \vert n \vert )a_{m}b_{n}}{\ln^{\lambda} \vert m \vert - \ln^{\lambda} \vert n \vert } \\&\quad< \frac{2\pi^{2}}{[\lambda\sin(\frac{\pi\lambda_{1}}{\lambda} )]^{2}} \Biggl[ \sum_{ \vert m \vert = 2}^{\infty} \frac{\ln^{p(1 - \lambda_{1}) - 1} \vert m \vert }{ \vert m \vert ^{1 - p}}a_{m}^{p} \Biggr]^{\frac{1}{p}} \Biggl[ \sum_{ \vert n \vert = 2}^{\infty} \frac{\ln^{q(1 - \lambda_{2}) - 1} \vert n \vert }{ \vert n \vert ^{1 - q}}b_{n}^{q} \Biggr]^{\frac{1}{q}}.\end{aligned} $$

It follows that (20) is an extension of (31). In particular, for $\lambda= 1$, $\lambda_{1} = \frac{1}{q}$, $\lambda_{2} = \frac{1}{p} $, we have the following simple Mulholland-type inequality in the whole plane with the best possible constant factor $\frac{2\pi^{2}}{\sin^{2}(\frac{\pi}{p})} $:

32 $$ \sum_{ \vert n \vert = 2}^{\infty} \sum _{ \vert m \vert = 2}^{\infty} \frac{\ln(\ln \vert m \vert /\ln \vert n \vert )}{\ln( \vert m \vert / \vert n \vert )} a_{m}b_{n} < \frac{2\pi^{2}}{\sin^{2}(\frac{\pi}{p})} \Biggl( \sum_{ \vert m \vert = 2}^{\infty} \frac{a_{m}^{p}}{ \vert m \vert ^{1 - p}} \Biggr)^{\frac{1}{p}} \Biggl( \sum _{ \vert n \vert = 2}^{\infty} \frac{b_{n}^{q}}{ \vert n \vert ^{1 - q}} \Biggr)^{\frac{1}{q}}. $$

(ii) If $a_{ - m} = a_{m}$, $b_{ - n} = b_{n}$ ($m,n \in\mathbf{N}\setminus \{ 1\} $), then (20) reduces to

33 $$ \begin{aligned}[b] &\sum_{n = 2}^{\infty} \sum_{m = 2}^{\infty} \biggl\{ \frac{\ln[\ln(m - \xi)/\ln(n - \eta)]}{\ln^{\lambda} (m - \xi) - \ln^{\lambda} (n - \eta)} + \frac{\ln[\ln(m - \xi)/\ln(n + \eta )]}{\ln^{\lambda} (m - \xi) - \ln^{\lambda} (n + \eta)} \\ &\qquad{} + \frac{\ln[\ln(m + \xi)/\ln(n - \eta)]}{\ln ^{\lambda} (m + \xi ) - \ln^{\lambda} (n - \eta)} + \frac{\ln[\ln(m + \xi)/\ln(n + \eta )]}{\ln^{\lambda} (m + \xi) - \ln^{\lambda} (n + \eta)} \biggr\} a_{m}b_{n} \\ &\quad< \frac{2\pi^{2}}{[\lambda\sin(\frac{\pi\lambda_{1}}{\lambda} )]^{2}} \Biggl\{ \sum_{m = 2}^{\infty} \biggl[ \frac{\ln^{p(1 - \lambda _{1}) - 1}(m - \xi)}{(m - \xi)^{1 - p}} + \frac{\ln^{p(1 - \lambda_{1}) - 1}(m + \xi)}{(m + \xi)^{1 - p}} \biggr]a_{m}^{p} \Biggr\} ^{\frac{1}{p}} \\ &\qquad{}\times \Biggl\{ \sum_{n = 2}^{\infty} \biggl[ \frac{\ln^{q(1 - \lambda_{2}) - 1}(n - \eta)}{(n - \eta)^{1 - q}} + \frac{\ln^{q(1 - \lambda_{2}) - 1}(n + \eta)}{(n + \eta)^{1 - q}} \biggr]b_{n}^{q} \Biggr\} ^{\frac{1}{q}}. \end{aligned} $$

In particular, for $\lambda= 1$, $\lambda_{1} = \frac{1}{q}$, $\lambda_{2} = \frac{1}{p}$, $\xi= \eta\in[0,\frac{1}{2}] $, we obtain

34 $$ \begin{aligned}[b]& \sum_{n = 2}^{\infty} \sum_{m = 2}^{\infty} \biggl\{ \frac{\ln[\ln(m - \xi)/\ln(n - \xi)]}{\ln[(m - \xi)/(n - \xi)]} + \frac{\ln[\ln(m - \xi)/\ln(n + \xi)]}{\ln[(m - \xi)/(n + \xi)]} \\ &\qquad{} + \frac{\ln[\ln(m + \xi)/\ln(n - \xi)]}{\ln[(m + \xi)/(n - \xi)]} + \frac{\ln[\ln(m + \xi)/\ln(n + \xi)]}{\ln[(m + \xi)/(n + \xi)]} \biggr\} a_{m}b_{n} \\ &\quad< \frac{2\pi^{2}}{\sin^{2}(\frac{\pi}{p})} \Biggl\{ \sum_{m = 2}^{\infty} \biggl[ \frac{1}{(m - \xi)^{1 - p}} + \frac{1}{(m + \xi)^{1 - p}} \biggr]a_{m}^{p} \Biggr\} ^{\frac{1}{p}}\\&\quad{}\times \Biggl\{ \sum_{n = 2}^{\infty} \biggl[ \frac{1}{(n - \xi)^{1 - q}} + \frac{1}{(n + \xi)^{1 - q}} \biggr]b_{n}^{q} \Biggr\} ^{\frac{1}{q}}. \end{aligned} $$

For $\xi= 0 $, (34) reduces to the following simple Mulholland-type inequality with the best possible constant factor $\frac{\pi^{2}}{\sin^{2}(\frac{\pi}{p})} $:

35 $$ \sum_{n = 2}^{\infty} \sum _{m = 2}^{\infty} \frac{\ln(\ln m/\ln n)}{\ln (m/n)} a_{m}b_{n} < \frac{\pi^{2}}{\sin^{2}(\frac{\pi}{p})} \Biggl( \sum_{m = 2}^{\infty} \frac{a_{m}^{p}}{m^{1 - p}} \Biggr)^{\frac{1}{p}} \Biggl( \sum _{n = 2}^{\infty} \frac{b_{n}^{q}}{n^{1 - q}} \Biggr)^{\frac{1}{q}}. $$

## Conclusions

In this paper, we present a new discrete Mulholland-type inequality in the whole plane with a best possible constant factor that is similar to that in (4) via introducing multi-parameters, applying weight coefficients, and using Hermite–Hadamard’s inequality in Theorem 1 and Theorem 2. Moreover, the equivalent forms, some particular cases, and the operator expressions are considered. The lemmas and theorems provide an extensive account of this type of inequalities.
